# Disease burden of appendectomy for appendicitis: a population-based cohort study

**DOI:** 10.1007/s00464-019-06738-6

**Published:** 2019-03-27

**Authors:** Elisabeth M. L. de Wijkerslooth, Anne Loes van den Boom, Bas P. L. Wijnhoven

**Affiliations:** Department of Surgery, Erasmus MC - University Medical Center, PO Box 2040, 3000CA Rotterdam, The Netherlands

**Keywords:** Appendicitis, Appendectomy, Length of stay, Hospital costs

## Abstract

**Background:**

Few large-scale epidemiologic studies evaluate the clinical and economic burden of appendicitis. These data may impact future research and treatment strategies. In this study, the objective was to determine the burden of appendectomy for appendicitis in terms of incidence rates, length of hospital stay (LOS) and hospital costs on a national level. In addition, outcomes were compared for subgroups based on surgical treatment, age and hospital setting.

**Methods:**

Observational retrospective population-based cohort study using the national Dutch healthcare reimbursement registry, which covers hospital registration and reimbursement for 17 million inhabitants. Patients with a diagnosis of appendicitis who underwent appendectomy between 2006 and 2016 were included. Primary outcomes were incidence rates, LOS and hospital costs.

**Results:**

A total of 135,025 patients were included. Some 53% of patients was male, and 64% was treated in a general hospital. The overall incidence rate of appendectomy was 81 per 100,000 inhabitants and showed a significant decreasing trend across time and age. Mean ± SD LOS per patient was 3.66 ± 3.5 days. LOS showed a significant increase with age and was significantly longer for open versus minimally invasive appendectomy. Mean ± SD hospital costs per patient were €3700 ± 1284. Costs were initially lower for open compared to minimally invasive appendectomy, but were similar from 2012 onward. Compared to non-university hospitals, patients treated in university hospitals had a significantly longer LOS and higher costs.

**Conclusions:**

Appendectomy for appendicitis represents a substantial clinical and economic burden in the Netherlands. A preference for minimally invasive technique seems justified.

Although acute appendicitis is highly prevalent among adults and children worldwide, literature on the clinical and economic burden of the disease is scarce. Emergent appendectomy remains the cornerstone of treatment and is nowadays mostly performed via the minimally invasive approach in Western countries [1–3]. It is known as a low-risk surgical procedure, with reported mortality rates between 0.03 and 0.24% [4–6]. Depending on the intraoperative classification, patients may be discharged within 24–48 h, or after a few days of prolonged antibiotic prophylaxis [7, 8]. Infectious complications occur in some 9–20% of patients, accompanied by a hospital readmission rate of 6% [5, 9]. Whereas morbidity and mortality are estimators of the burden of disease in a population, the economic burden should also be taken into account. Data on the hospital costs related to appendicitis may impact future treatment and research strategies. This is especially relevant in light of the increasing interest in the non-operative treatment approach [10–14]. Apart from avoiding surgery and its potential complications, non-operative treatment might also be beneficial in terms of healthcare cost savings. However, the available evidence is ambiguous [15–18]. Regarding the choice of operative approach, most studies have demonstrated comparable or better clinical outcomes for minimally invasive compared to open appendectomy, however at higher medical care costs [19–22].

Several population-based studies on the incidence of appendicitis have been published, as recently summarized in a systematic review on the global incidence of appendicitis [23]. Fewer large-scale studies have taken the economic burden of appendicitis into account [24–27]. No study to our knowledge has yet simultaneously evaluated both the clinical and financial burden of appendicitis and appendectomy on a population-level.

The primary aim of this study was to evaluate the burden of appendectomy for appendicitis in the Netherlands in terms of hospital costs, length of hospital stay and incidence. Secondary aims were to evaluate outcomes according to surgical approach, registration year, age and hospital setting, and explore trends.

## Materials and methods

### Study design and setting

The present study was a population-based retrospective observational cohort study based on the national healthcare reimbursement system, which contains data from all hospitals and medical facilities in the Netherlands. The study protocol was reviewed and approved by the Erasmus MC Ethics Committee. Requirement for informed consent was waived, owing to the observational and anonymous nature of this study.

### Database

Hospital reimbursement by means of Diagnosis Related Groups (DRGs) has become common worldwide. Since 2005, medical care registration and reimbursement in the Netherlands is performed through a DRG-like case-mix system based on diagnosis treatment combinations (DBCs). A DBC contains the complete set of care activities required to establish a particular diagnosis and treatment, from first presentation to the hospital up to the last check-up [28]. DBC registration is collected in a national healthcare database: the so-called DBC Information System (DIS). All data relevant for reimbursement is registered (diagnoses, treatment activities, hospital setting, length of stay) as well as a limited number of patient characteristics. Detailed data such as type of appendicitis, complications and readmission cannot be retrieved from the DIS. The database is managed by the Dutch Healthcare Authority (NZa), an autonomous administrative authority that is part of the Dutch Ministry of Health, Welfare and Sport. For the current study, data was extracted and aggregated by the NZa, as available per March 1, 2018. Subsequent analyses were performed by the authors.

### Case selection

The DIS database was queried for all patients registered with a diagnosis of appendicitis that underwent appendectomy between 2005 and 2016, as from 2016 onward the registration was not complete yet. Appendicitis was identified using specialist-diagnosis codes for appendicitis belonging to medical specializations surgery (0303; 113) and pediatrics (0316; 3302). Appendectomy was identified via specific care activity codes for open appendectomy (034910) and minimally invasive appendectomy (034911). Patients that had other surgical procedures of the appendix (i.e., periappendiceal abscess surgery or synchronous cholecystectomy and appendectomy) were excluded from the present analysis. The data for 2005 reflected less dependable registration during the starting year of the DIS database. Hence, a choice was made to limit the final case selection to January 2006–December 2015 for the most valid analysis.

### Collected data

Data were collected on year of presentation, gender, age, hospital setting (university hospital, top-clinical hospital, general hospital), surgical procedure (open or minimally invasive appendectomy), length of hospital stay (LOS) and hospital costs. LOS concerns the duration of the admission from first presentation to the hospital until discharge after surgery. Admission days related to readmission(s) are not included in the same DBC. Hospital costs were calculated based on reimbursements per DBC by the hospitals in the DIS-system. Each specific DBC has a fixed price—either nationally standardized or negotiated upon between health insurers and hospitals—which covers both direct medical costs and specialists’ fees. Cases representing the lowest and highest 10% of reimbursed costs were excluded from cost analysis, as per standard NZa-policy.

### Outcome measures

The outcome measures in this study are: incidence (per 100,000 inhabitants), LOS (in days) and direct hospital costs (in euros). Outcomes were stratified by year of DBC registration, age and hospital setting. Dutch population statistics were retrieved from the electronic databank Statline, managed by Statistics Netherlands (CBS).

### Statistical analysis

Outcomes are reported using descriptive statistics. Incidence rates are presented as number per 100,000 inhabitants. Categorical outcomes are presented as no. of cases (%) and continuous outcomes as means ± standard deviations (SD) as well as medians and interquartile ranges (IQR). The Student t test and Chi-square test were used to compare means and proportions, as appropriate. Furthermore, the Cochrane-Armitage test was used to evaluate trends in incidence and proportion of minimally invasive surgery over time (per registration year) and age group (per decade). A value of *p* < 0.05 was considered significant. The Holm method was used to correct for multiple testing [29]. Adjusted *p* values are reported. Data analysis was performed using Excel 2010 (Microsoft, Redmond, Washington, USA), SPSS version 21 (IBM, Armonk, New York, USA) and R version 3.5.1 (Feather Spray Package; https://cran.r-project.org/).

This manuscript was written using the Strengthening the Reporting of OBservational studies in Epidemiology (STROBE) statement checklist [30].

## Results

### Study population

**Fig. 1:**
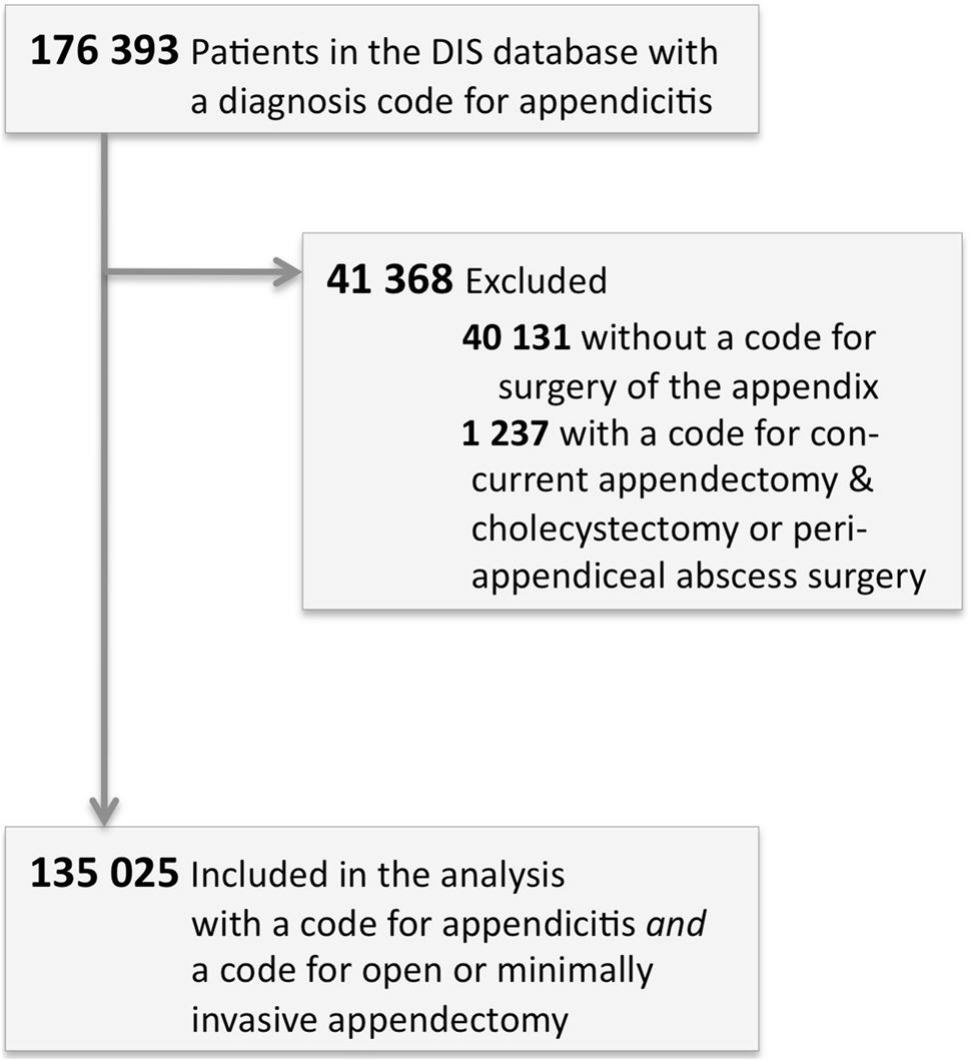
Study flowchart. Subscript: another 20,442 patients had a registered surgical procedure of the appendix but no registered diagnosis of appendicitis (13% of all 156,704 patients with a surgical procedure of the appendix within the study timeframe)

A total of 135,025 patients met the inclusion criteria (Fig. 1). Basic patient characteristics and outcomes are shown in Table 1. The proportion of minimally invasive appendectomy was lower for men compared to women (42% vs. 60%, *p* < 0.0001). Over time the proportion of patients operated minimally invasive increased (Table 2). This trend was statistically significant (*p* < 0.0001) for the total study population as well as for male and female patients separately.

**Table 1:** Characteristics and outcomes of patients with appendicitis and appendectomy 2006–2015

Variable	No. (%)	Incidence^a^	Length of stay in days			Hospital costs in euros		
			Mean ± SD	*p* value	Median (IQR)	Mean ± SD	*p* value	Median (IQR)
Total	135 025	81	3.66 ± 3.52		3 (2–4)	3700 ± 1284		3645 (3350–4095)
Sex								
Male	71 054 (53)	86	3.71 ± 3.63	< 0.0001	3 (2–4)	3680 ± 1313	< 0.0001	3580 (3350–4075)
Female	63 971 (47)	76	3.60 ± 3.40		3 (2–4)	3723 ± 1250		3720 (3390–4125)
Surgical approach								
Open	67 444 (50)	41	3.83 ± 3.82	< 0.0001	3 (2–5)	3584 ± 1320	< 0.0001	3455 (3280–3905)
Minimally invasive	68 067 (50)	41	3.49 ± 3.21		2 (2–4)	3817 ± 1242		3850 (3555–4125)
Hospital setting^b^								
UMC	6 933 (5)		4.49 ± 4.83	< 0.0001	3 (2–6)	4244 ± 2141	< 0.0001	4030 (3555–4925)
Top-clinical^c^	41 955 (31)		3.66 ± 3.55		2 (2–4)	3820 ± 1232		3850 (3450–4130)
				< 0.01			< 0.0001	
General	86 104 (64)		3.59 ± 3.37		3 (2–4)	3598 ± 1198		3555 (3315–4030)

*SD* standard deviation, *IQR* interquartile range, *UMC* university medical center

^a^Incidence rates are presented per 100,000 inhabitants

^b^33 remaining patients were treated in a hospital setting other than UMC, top-clinical or general hospital (i.e. private clinic)

^c^Top-clinical centers are non-academic hospitals that provide more complex care than general hospitals and usually have an important role in training doctors and in conducting scientific research

**Table 2:** Outcomes according to surgical approach, year of registration and age group

Subgroup	No. (%)^a^	Incidence^b^	Length of stay in days		Hospital costs in euros	
			Mean ± SD	Median (IQR)	Mean ± SD	Median (IQR)
By year of registration and surgical approach						
All cases						
2006	14 651 (11)	90	4.08 ± 4.45	3 (2–5)	4517 ± 1951	4950 (4325–5510)
2007	14 161 (11)	87	3.75 ± 3.77	3 (2–5)	3061 ± 1054	3085 (2720–3640)
2008	13 851 (10)	84	3.91 ± 3.57	3 (2–5)	3636 ± 476	3455 (3450–4030)
2009	14 015 (10)	85	3.75 ± 3.40	3 (2–4)	3835 ± 503	3725 (3555–4125)
2010	13 179 (10)	80	3.69 ± 3.47	3 (2–4)	3436 ± 453	3430 (3280–3555)
2011	12 516 (9)	75	3.46 ± 3.25	2 (2–4)	3534 ± 776	3850 (3350–3850)
2012	12 615 (9)	75	3.37 ± 3.36	2 (2–4)	3708 ± 1433	3525 (3030–3980)
2013	12 754 (10)	76	3.53 ± 3.26	2 (2–4)	3886 ± 1261	3710 (3475–4070)
2014	14 137 (11)	84	3.46 ± 3.08	2 (2–4)	3776 ± 1399	3850 (3510–4155)
2015	13 146 (10)	78	3.50 ± 3.23	2 (2–4)	3556 ± 1796	3860 (3565–4115)
Open procedures						
2006	11 016 (75)		4.17 ± 4.67**	3 (2–5)	4423 ± 1945***	4865 (4275–5410)
2007	10 007 (71)		3.80 ± 3.87	3 (2–5)	2853 ± 964***	2905 (2645–3215)
2008	9 044 (65)		4.06 ± 3.79***	3 (2–5)	3438 ± 424***	3455 (3440–3455)
2009	8 178 (58)		3.85 ± 3.57**	3 (2–4)	3590 ± 437***	3555 (3555–3555)
2010	6 778 (51)		3.77 ± 3.56*	3 (2–4)	3275 ± 408***	3280 (3260–3280)
2011	5 672 (45)		3.54 ± 3.38	2 (2–4)	3260 ± 685***	3350 (3350–3370)
2012	5 104 (41)		3.52 ± 3.64**	2 (2–4)	3733 ± 1495	3530 (3105–3980)
2013	4 381 (34)		3.70 ± 3.53**	2 (2–5)	3906 ± 1407	3705 (3460–4040)
2014	4 133 (29)		3.58 ± 3.27*	2 (2–4)	3833 ± 1560*	3835 (3530–4115)
2015	3 131 (24)		3.80 ± 3.52***	2 (2–5)	3613 ± 2119	3905 (3570–4115)
Minimally invasive						
2006	3 693 (25)		3.85 ± 3.76**	3 (2–5)	4794 ± 1939***	5105 (4520–5835)
2007	4 239 (30)		3.63 ± 3.53	3 (2–4)	3563 ± 1088***	3680 (3245–4075)
2008	4 886 (35)		3.65 ± 3.14***	3 (2–4)	4007 ± 319***	4030 (4030–4030)
2009	5 942 (42)		3.61 ± 3.16**	3 (2–4)	4176 ± 372***	4125 (4125–4125)
2010	6 455 (49)		3.61 ± 3.36*	2 (2–4)	3606 ± 433***	3555 (3510–3630)
2011	6 881 (55)		3.41 ± 3.14	2 (2–4)	3760 ± 774***	3850 (3850–3850)
2012	7 532 (60)		3.27 ± 3.15**	2 (2–4)	3692 ± 1390	3510 (3030–3980)
2013	8 395 (66)		3.45 ± 3.11**	2 (2–4)	3875 ± 1176	3715 (3495–4095)
2014	10 013 (71)		3.41 ± 3.00*	2 (2–4)	3753 ± 1326*	3870 (3490–4160)
2015	10 031 (76)		3.42 ± 3.16***	2 (2–4)	3542 ± 1719	3830 (3550–4120)
By age group and surgical approach						
All cases						
0–9	10 237 (8)	54	3.79 ± 3.57	3 (2–5)	3652 ± 1401	3555 (3300–4085)
10–19	36 466 (27)	182	3.40 ± 3.13	2 (2–4)	3698 ± 1299	3620 (3350–4085)
20–29	25 595 (19)	126	3.08 ± 2.65	2 (2–3)	3709 ± 1170	3715 (3390–4125)
30–39	19 652 (15)	90	3.28 ± 2.99	2 (2–4)	3710 ± 1270	3690 (3370–4125)
40–49	16 444 (12)	64	3.66 ± 3.21	3 (2–5)	3710 ± 1218	3675 (3360–4115)
50–59	12 703 (9)	55	4.20 ± 4.04	3 (2–5)	3709 ± 1303	3650 (3350–4085)
60–69	8 363 (6)	45	4.72 ± 4.72	4 (2–6)	3700 ± 1379	3620 (3350–4075)
70–79	4 038 (3)	36	5.77 ± 5.61	4 (2–7)	3707 ± 1529	3615 (3350–4080)
≥ 80	1 527 (1)	23	7.53 ± 6.99	6 (3–9)	3627 ± 1373	3555 (3280–4030)
Open procedures						
0–9	7 654 (75)		3.76 ± 3.52	3 (2–5)	3573 ± 1387	3485 (3485–3910)
10–19	19 324 (53)		3.45 ± 3.18	3 (2–4)	3575 ± 1287	3455 (3280–3870)
20–29	10 511 (41)		3.16 ± 2.70	2 (2–4)	3566 ± 1216	3455 (3280–3900)
30–39	8 749 (45)		3.41 ± 3.11	3 (2–4)	3557 ± 1318	3455 (3280–3925)
40–49	7 560 (46)		3.86 ± 3.49	3 (2–5)	3597 ± 1291	3460 (3280–3980)
50–59	6 142 (48)		4.49 ± 4.58	3 (2–6)	3619 ± 1381	3485 (3280–3950)
60–69	4 268 (51)		5.01 ± 5.36	4 (2–6)	3646 ± 1446	3485 (3280–3915)
70–79	2 252 (56)		6.21 ± 6.07	5 (3–8)	3640 ± 1454	3490 (3280–3970)
≥ 80	984 (64)		8.13 ± 7.59	6 (4–10)	3591 ± 1436	3455 (3245–3970)
Minimally invasive						
0–9	2 597 (25)		3.89 ± 3.69	3 (2–5)	3891 ± 1419	3960 (3555–4185)
10–19	17 255 (47)		3.34 ± 3.08	2 (2–4)	3838 ± 1322	3850 (3555–4125)
20–29	15 169 (59)		3.03 ± 2.63	2 (2–3)	3809 ± 1126	3850 (3555–4125)
30–39	10 978 (56)		3.20 ± 2.93	2 (2–4)	3834 ± 1215	3850 (3555–4125)
40–49	8 977 (55)		3.50 ± 2.94	2 (2–4)	3804 ± 1144	3850 (3550–4125)
50–59	6 614 (52)		3.94 ± 3.47	3 (2–5)	3792 ± 1218	3850 (3535–4125)
60–69	4 130 (49)		4.44 ± 3.92	3 (2–6)	3758 ± 1301	3850 (3510–4125)
70–79	1 799 (45)		5.24 ± 4.91	4 (2–6)	3796 ± 1614	3840 (3515–4125)
≥ 80	548 (36)		6.47 ± 5.64	5 (3–8)	3687 ± 1250	3850 (3510–4125)

*SD* standard deviation, *IQR* interquartile range

*p < 0.05, **p < 0.001 and ***p < 0.0001 for the difference in outcome between open versus minimally invasive appendectomy

^a^Numbers of open and minimally invasive procedures may not add up to the total numbers in this column owing to 486 double procedure registries. Percentages have been rounded and may not total 100

^b^Incidence rates are presented per 100,000 inhabitants

### Incidence

**Fig. 2:**
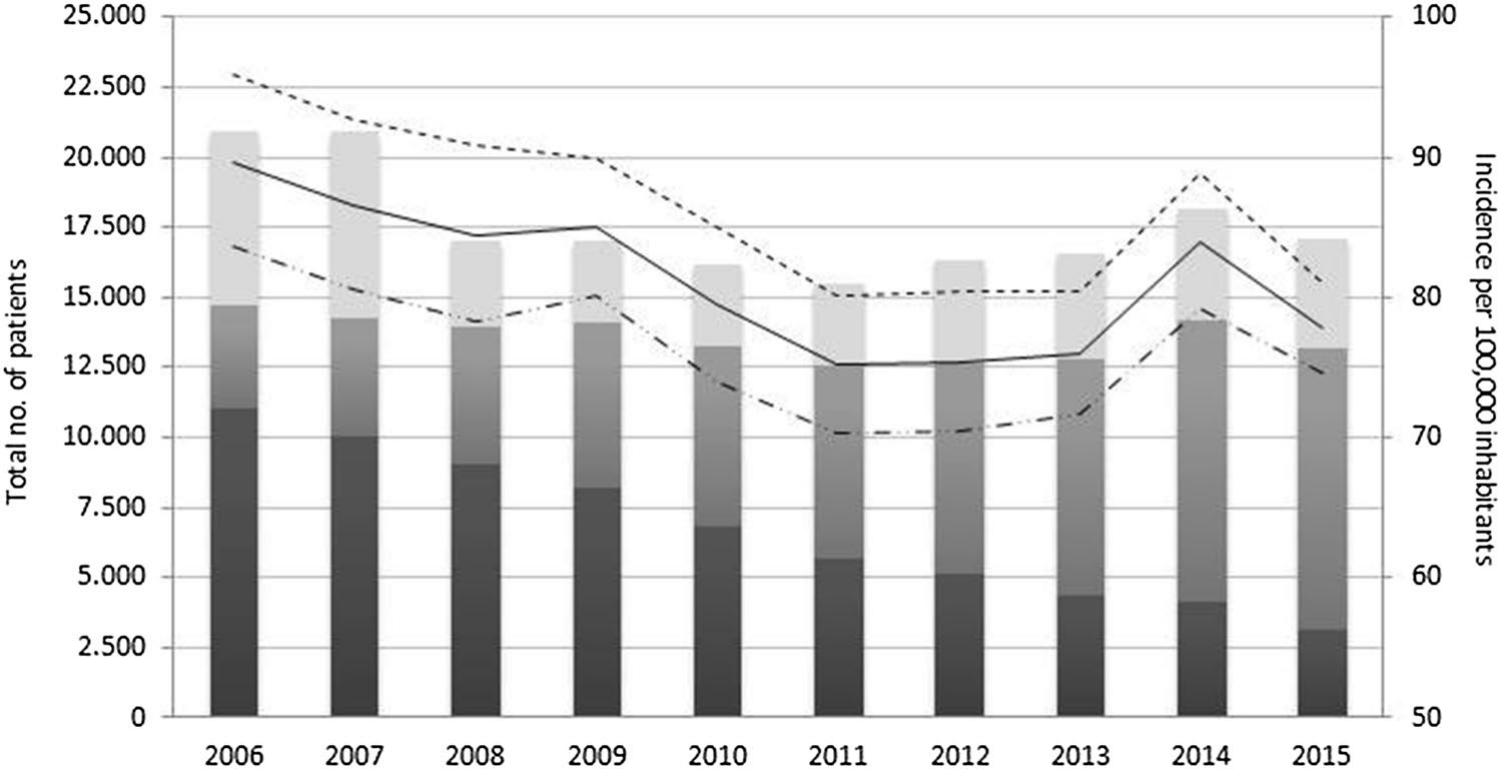
Absolute number and incidence rate of patients with appendicitis and appendectomy. Dark shaded grey bar: open appendectomy, medium shaded grey bar: minimally invasive appendectomy, light shaded grey bar: no surgery, solid line: incidence (all), dotted line: incidence men, dashed dotted line: incidence women

The overall incidence was 81 per 100,000 inhabitants (range 75–90). Incidence was higher for men compared to women (Table 1) and showed a decreasing trend over time (*p* < 0.0001) for the total study population (Table 2) and for men and women separately as well. Incidence was highest at 182 per 100 000 inhabitants aged 10–19 years. A decreasing trend across age groups (*p* < 0.0001) was observed toward 23 per 100 000 inhabitants aged ≥ 80 years (Table 2; Fig. 2).

### Length of hospital stay

**Fig. 3:**
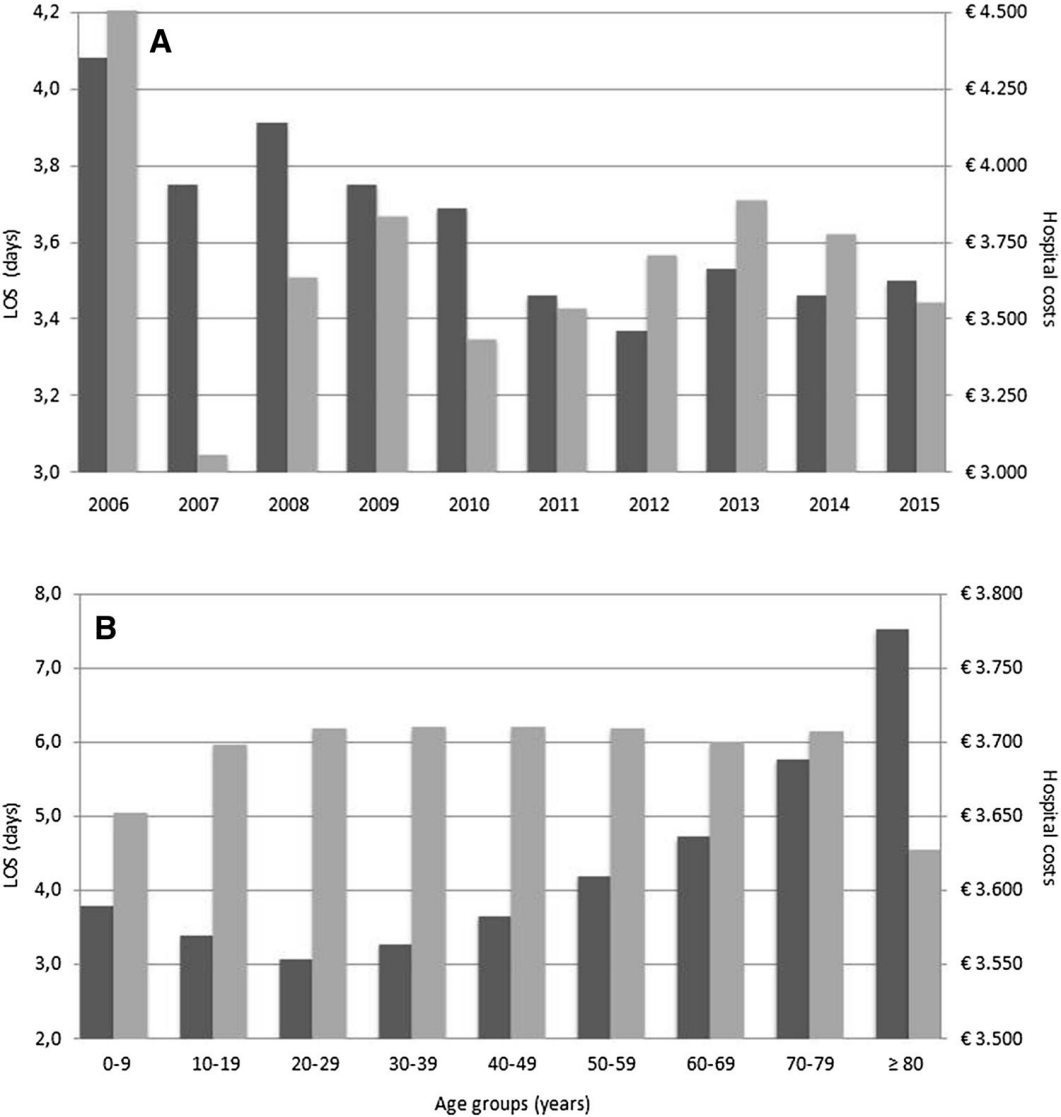
**A**. Mean LOS and hospital costs per patient according to year of registration. Dark shaded grey bar: mean LOS per patient, light shaded grey bar: Mean hospital costs per patient, subscript: *LOS* length of hospital stay. **B** Mean LOS and hospital costs per patient according to age group. Dark shaded grey bar: Mean LOS per patient, light shaded grey bar: mean hospital costs per patient. Subscript: *LOS* length of hospital stay

Some 127,942 patients (95%) had at least one registered day of hospital stay. Mean LOS ± SD per patient was 3.66 ± 3.52 days. The mean total number of admission days registered per year for patients undergoing appendectomy for appendicitis was 49,419. Mean ± SD LOS was shorter for minimally invasive compared to open surgery (3.49 ± 3.21 vs. 3.83 ± 3.82, *p* < 0.0001) as well as for general versus top-clinical hospitals (*p* < 0.01) and for top-clinical versus university hospitals (*p* < 0.0001): 3.6 ± 3.2 versus 3.7 ± 3.6 versus 4.5 ± 4.8 days, respectively (Table 1). Overall mean LOS decreased over time and from age group 30–39 years onward mean LOS gradually increased with age (Table 2; Fig. 3a, b).

### Hospital costs

Overall mean ± SD hospital costs were €3700 ± 1284 per patient, which corresponds to national annual mean of €49,959,250 during the study period. Costs were higher for patients who underwent minimally invasive versus open appendectomy (€3817 ± 1242 vs. €3584 ± 1320, *p* < 0.0001). Analysis per registration year demonstrated that the difference was significant from 2006 to 2011, but costs were in the same range or significantly lower for minimally invasive appendectomy from 2012 onward (Table 2). Mean costs per patient in university hospitals compared to top-clinical and general hospitals were €4244 versus €3820 versus €3598, respectively (*p* < 0.0001).

## Discussion

The present study demonstrates that the burden of appendectomy for appendicitis is substantial and implicates that treatment by means of minimally invasive appendectomy at a general hospital is most favorable. The incidence between 2006 and 2016 was 81 per 100,000 inhabitants. And mean length of stay and hospital costs were 3.66 days and €3700 per patient. This translates into approximately 13,500 patients annually that are responsible for nearly 50,000 admission days and close to 50 million euro of hospital costs. Minimally invasive appendectomy was consistently associated with shorter LOS compared to open surgery and with comparable hospital costs from 2012 onward. In addition, treatment in university hospitals resulted in significantly longer LOS and higher costs, compared to other hospital settings. However, it is important to point out that we were unable to correct for potential confounders in this analysis, which may have influenced the results (i.e., university hospitals may have treated patients with more comorbidities, which may have affected their recovery and length of stay).

The number of patients in this cohort is an underestimation of the total population with appendicitis, who underwent surgical *and* non-surgical treatment. Non-operative treatment for appendicitis and incidental appendectomies were excluded as it was impossible to identify these patients from the database. Twenty-three per cent of patients with a diagnosis of appendicitis did not have a surgical procedure linked and were excluded. Clearly, this cannot entirely be interpreted as non-operative treatment of appendicitis, since a much lower proportion would be expected given the time period that was selected. Non-operative treatment has just recently been advocated as an alternative to surgery, at least in the Netherlands. More likely, for some patients the (initial) diagnosis of appendicitis may have been wrong, given that no surgical treatment was registered. At the same time, a considerable number of patients with a surgical procedure of the appendix were excluded since a diagnosis of appendicitis was missing. It is doubtful that those patients all underwent incidental appendectomies. We consider it more likely that some of them underwent a diagnostic laparoscopy under suspicion of different pathology and turned out to have appendicitis, but afterward the DBC diagnosis was not adjusted. Taken together, the actual number of patients annually treated for appendicitis in the Netherlands may be higher than presented here.

A significant decrease in incidence was observed over the 10-year study period (from 90 per 100,000 in 2006 to 78 per 100,000 in 2015, trend test *p* < 0.0001). This may reflect a decrease in patients presenting with acute appendicitis and/or a decrease in the number of patients treated surgically. The decrease in incidence observed in this study seems to be in line with results from a recently published population-wide study among children in Sweden that demonstrated a significant decline in incidence of appendicitis over time [31]. Then again, a nationwide epidemiological study on appendicitis in the USA published in 2012 reported a significant increase in incidence between 1993 and 2008 [32]. The literature on incidence of appendicitis is not clear [31, 33–35]. In a systematic review on the global incidence of appendicitis, pooled incidence of appendicitis or appendectomy in the Western World was estimated at 151 per 100 000 person years and reported to be stable in most Western countries [23]. The finding in this cohort that incidence peaks among persons aged 10–19 years, and is greater among men compared to women, is consistent with previous epidemiological literature [1, 32, 36, 37]. In 2010, a new Dutch guideline on treatment of appendicitis was published, which incorporates ultrasound or CT imaging in the standard diagnostic process [38, 39]. It is plausible that fewer patients were operated as a result due to better diagnosis and a fall in the proportion of appendectomy for appendix sana. A large Dutch cohort study (*n* = 1943) performed in 2014 demonstrated a low negative appendectomy rate of 3% [38, 39]. Another factor that might play a role is the growing popularity of non-operative treatment and fading dogma ‘when in doubt, take it out’. With a growing number of papers presenting good results for the non-operative approach, surgeons may already be less inclined to take patients straight to theater [10–12, 40]. As discussed before, the DIS database does not allow for accurate identification of non-operative treatment of appendicitis. Neither does it contain information on histopathological examination of the appendices. Therefore, it is impossible to estimate the potential effect of a supposedly decreased negative appendectomy rate and increased non-operative treatment rate in this study.

The use of minimally invasive technique significantly increased over time. This seems justified since minimally invasive appendectomy was consistently associated with a shorter hospital stay and similar costs from 2012 onward. Patients were admitted to the hospital approximately 3.5 days in case of laparoscopic appendectomy and slightly (but significantly) longer in case of open surgery (3.8 days). This finding has been reported before in a national cohort from the USA for 2004–2011, as well as other studies [36]. Interestingly, for 5% of patients not one hospital admission day was registered, which may reflect a proportion of *same-day discharge* patients. Several recent studies have indicated that same-day discharge is safe after appendectomy for simple appendicitis. Both the proportion of minimally invasive surgery and same-day discharge can be expected to increase in the future, which may reduce costs. No clear trend in hospital costs was observed during the study period. Within the DIS registration, patients are categorized into three groups based on length of stay, which may explain that hospital costs do not seem to increase or decrease directly following changes in length of stay. It appears as though mean hospital costs fluctuated considerably in the early years of the DIS database and stabilized somewhat toward the end of the study period. Whereas from 2006 to 2011 hospital costs were significantly higher for patients that underwent minimally invasive appendectomy, from 2012 onward this was not the case anymore. Moreover, in 2014 the hospital costs were significantly higher for patients that underwent open appendectomy. This is of interest, since most previous studies demonstrated lower or comparable costs [19, 21, 41]. Furthermore, this study only evaluated differences in direct hospital costs, whereas there may likely be additional benefit of minimally invasive surgery in terms societal costs (i.e., faster recovery resulting in less sick leave) [41, 42]. With an abundance of evidence showing that a laparoscopic technique surpasses open surgery in clinical outcomes [22, 41, 43, 44], at similar cost as presented here, laparoscopic appendectomy should likely be the first-choice surgical approach.

In the Netherlands and several other Western countries, healthcare costs have risen over the past decades, with over 10% of the gross domestic product being spent on healthcare [45–47]. In general, long-term care for the elderly form the greater part of healthcare costs [45]. Nevertheless, acute appendicitis forms a substantial economic burden due to the large number of patients. This study indicates that appendicitis and appendectomy produced almost €47 million in hospital costs in 2015, which is 0.8% of the total €5915.6 million in hospital costs for diseases of the digestive tract in the same year according to the Central Bureau for Statistics in the Netherlands [48]. Wherever possible, the aim should be to reduce costs without compromising clinical outcomes. Based on the present results, treatment of appendicitis in general hospitals is preferable over treatment in top-clinical and university hospitals, both in terms of hospital costs and length of stay. A minor proportion (5%) of patients in the present cohort was treated in university hospitals. Presumably these patients represent a selected sample of more complex, high-risk patients and therefore require longer hospital admission and higher cost of care compared to patients in other hospital settings. Differences in length of stay and hospital costs between top-clinical hospitals and general hospitals were smaller, yet significant. In this cohort, already the majority of cases (64%) was treated in general hospitals and, assuming equivalent outcomes, this may be further encouraged. Non-operative treatment of appendicitis has also been proposed to be an economical choice [17], at no compromise in safety according to several recent studies [11, 49, 50]. Patients treated non-operatively were excluded from this study, and the DBC reimbursement system does not allow for discrimination of operating costs from admission day costs. Hence, no direct conclusions can be drawn with regard to the cost of operative versus non-operative management of acute appendicitis based on this cohort.

### Limitations and strengths

Some important limitations to this cohort study should be acknowledged. First, in a large administrative database like the DIS database some level of erroneous registration and miscoding may occur. The finding of a fairly large proportion of patients with a registered diagnosis of appendicitis without a surgical procedure of the appendix may be an indicator of this. Secondly, we were unable to further discriminate (and correct for) relevant potential confounders such as comorbidities, the type of appendicitis (simple/complex) and postoperative complications, which are known to influence length of stay and hospital costs. Unfortunately, the DIS-database does not contain all these parameters. Nevertheless, the main strength of the study is that we were able to analyze data from all Dutch hospitals in a nationwide cohort. And despite its limitations, we believe the present study provides an adequate estimation of the substantial burden of appendicitis and appendectomy.
